# ProCarDB: a database of bacterial carotenoids

**DOI:** 10.1186/s12866-016-0715-6

**Published:** 2016-05-26

**Authors:** L. N. U. Nupur, Asheema Vats, Sandeep Kumar Dhanda, Gajendra P. S. Raghava, Anil Kumar Pinnaka, Ashwani Kumar

**Affiliations:** Microbial Type Culture collection and Gene Bank (MTCC), Council of Scientific and Industrial Research, Institute of Microbial Technology, Chandigarh, India; Infectious Diseases and Immunity, Council of Scientific and Industrial Research, Institute of Microbial Technology, Chandigarh, India; Bioinformatics Centre, Council of Scientific and Industrial Research- Institute of Microbial Technology, Chandigarh, India; Microbial Type Culture collection and Gene Bank (MTCC), Council of Scientific and Industrial Research, Institute of Microbial Technology, Sector 39 A, 160036 Chandigarh, India; Infectious Diseases and Immunity, Council of Scientific and Industrial Research, Institute of Microbial Technology, Sector 39 A, 160036 Chandigarh, India

**Keywords:** Carotenoids, Carotenoid database, ProCarDB, Carotenoid biosynthesis pathways, Genes encoding for carotenoid biosynthesis, Applications of carotenoids, Identification of carotenoids

## Abstract

**Background:**

Carotenoids have important functions in bacteria, ranging from harvesting light energy to neutralizing oxidants and acting as virulence factors. However, information pertaining to the carotenoids is scattered throughout the literature. Furthermore, information about the genes/proteins involved in the biosynthesis of carotenoids has tremendously increased in the post-genomic era. A web server providing the information about microbial carotenoids in a structured manner is required and will be a valuable resource for the scientific community working with microbial carotenoids.

**Results:**

Here, we have created a manually curated, open access, comprehensive compilation of bacterial carotenoids named as ProCarDB- Prokaryotic Carotenoid Database. ProCarDB includes 304 unique carotenoids arising from 50 biosynthetic pathways distributed among 611 prokaryotes. ProCarDB provides important information on carotenoids, such as 2D and 3D structures, molecular weight, molecular formula, SMILES, InChI, InChIKey, IUPAC name, KEGG Id, PubChem Id, and ChEBI Id. The database also provides NMR data, UV-vis absorption data, IR data, MS data and HPLC data that play key roles in the identification of carotenoids. An important feature of this database is the extension of biosynthetic pathways from the literature and through the presence of the genes/enzymes in different organisms. The information contained in the database was mined from published literature and databases such as KEGG, PubChem, ChEBI, LipidBank, LPSN, and Uniprot. The database integrates user-friendly browsing and searching with carotenoid analysis tools to help the user. We believe that this database will serve as a major information centre for researchers working on bacterial carotenoids.

## Background

Carotenoids are the isoprenoid derivatives that are characterized by the presence of eight isoprenoids (C_5_) with a characteristic polyene chain of conjugated double bonds (CDB). The CDB result in distinguishing absorption patterns and are responsible for imparting colours and antioxidant activity to carotenoids [1]. Carotenoids are found in all the photosynthetic organisms as well as in a variety of non-photosynthetic organisms [2]. They perform a variety of functions, ranging from harvesting solar-energy [3, 4] to serve as antioxidants [5, 6] to radio-resistance [7] and as virulence factors in several pathogens [8–10]. Carotenoids are also known for modulating immune system [11], scavenging free-radicals [12], protection from UV radiations, Pro-vitamin A activity and other beneficial properties [13–15] and thus are exploited for the pharmaceutical purposes in many diseases as well as for the health maintenance. Lycopene, β-carotene, canthaxanthin, zeaxanthin and astaxanthin are commercially exploited by nutraceutical industry. Their importance as nutraceuticals is due to their cancer-preventive capabilities [16–20], regulation of lipid metabolism [21], antioxidant function [22], obesity prevention [23, 24], and increased longevity [25]. They are used in food products, cosmetics, vitamin supplements, health products as well as feed additives for poultry, livestock, fish and crustaceans [26]. The current demand for carotenoids as dietary supplements is estimated to be worth more than $1.2 billion (http://www.bccresearch.com/market-research/food-and-beverage/carotenoids-global-market-fod025d.html). These supplements are required because human and other animals cannot synthesize carotenoids and therefore, must acquire them through the diet. Their pharmacological importance is primarily attributed to their anti-cancerous/cancer preventive activity [18, 27, 28], anti-diabetic properties [16, 29] and the prevention of cardiovascular diseases though their roles as antioxidants [30, 31]. Given the diverse roles played by these carotenoids, the attention has been focused on the identification and isolation of microbes producing carotenoids and their biosynthetic pathways. Recent efforts have dissected the genetic pathways involved in the synthesis of carotenoids [32–34]. Understanding these pathways at the molecular level has allowed the use of genetic engineering approaches for the biosynthesis of known and novel carotenoids in model microbial strains [32, 35–37]. The knowledge of metabolic engineering and genetic engineering has been strengthened from the high demand for carotenoids as dietary supplements [38].

The literature reveals a vast body of information on different aspects of carotenoids, such as the abundance of microbial carotenoids, their chemical properties, methods of their characterization, the genetic networks that synchronize carotenoid synthesis, the metabolic pathways involved in the carotenoid biosynthesis, their pharmacological properties and other relevant applications. Despite the availability of this huge amount of information, a unique portal providing all the information at one place has been lacking. Some databases providing limited information on carotenoid consumption in European countries [39] or the carotenoid content in food [40] does exist. However, these databases lack information on microbial carotenoids and provide information on specific aspects of carotenoids in the food. In addition to these databases, information on carotenoids can be extracted from LipidBank (http://LipidBank.jp/) and PubChem (https://pubchem.ncbi.nlm.nih.gov/), and information on the pathways involved in carotenoid metabolism can be gathered from the Kyoto Encyclopedia of Genes and Genomes (KEGG, http://www.genome.jp/kegg/).

In the light of renewed interest in microbial carotenoids and the amount of information available on carotenoids, here we have created a user friendly database named ProCarDB (http://bioinfo.imtech.res.in/servers/procardb/) that organizes and provides all the information pertaining to identification, characterization and biosynthesis of carotenoids at one site.

## Construction and content

### Source of data

The data for ProCarDB were mined from the published literature and various databases. The keywords used for the literature search were “carotenoid,” “microbial carotenoid”, “bacterial carotenoids”, “pigmented bacteria”, and “prokaryotic carotenoid” on various data search platforms, including Pubmed, Google Scholar, International Journal of Systematic and Evolutionary Microbiology (IJSEM), Science Direct and Springer links. We have also consulted books such as Bergey’s Manual of Systematic Bacteriology (2nd edition, Bergey’s Manual Trust), Photosynthesis (J. Amesz, volume 15, 1987, published by Elsevier), Anoxygenic Photosynthetic Bacteria (R.E. Blankenship, M.T. Madigan, C.E. Bauer, 1995, Kluwer Academic Publishers), The Photochemistry of Carotenoids (H.A. Frank et al., 2004, Kluwer Academic Publishers) and Purple Phototrophic Bacteria (C.N. Hunter, F. Daldal, M.C. Thurnauer, J.T. Beatty, 2009, Springer) for harvesting the relevant information on carotenoids. The carotenoid data, such as structure, molecular formula, molecular weight, IUPAC name, InChI, SMILES, UV Spectra, NMR Spectra, and HPLC data were primarily compiled from original manuscripts published or from LipidBank [41]. We have also retrieved information from databases such as PubChem (https://pubchem.ncbi.nlm.nih.gov/), Chemical Entities of Biological Interest (ChEBI, www.ebi.ac.uk/chebi), LipidBank (http://LipidBank.jp/), chemspider (www.chemspider.com/), KEGG (http://www.genome.jp/kegg/) and Biopath (https://webapps.molecular-networks.com/biopath3/).

### Database architecture and web interface

**Fig. 1:**
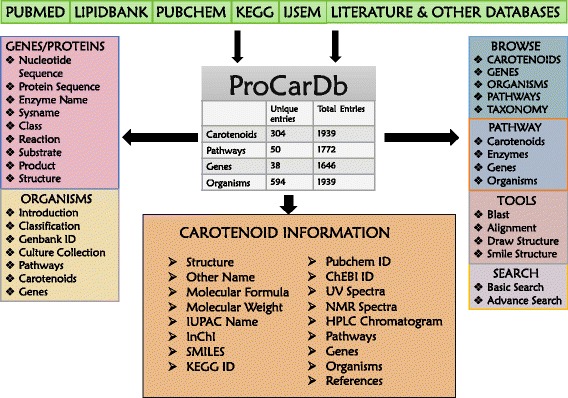
Architecture of ProCarDb Database

The architecture of the database is summarised in the Fig. 1. These data were integrated in MySQL. The Web interface was built in PHP, HTML, Javascript and CSS at the front end. This database can also be browsed from mobile phones or other hand-held devices.

### Integration of web tools/data access

#### Basic search and advanced search

The basic search is meant for users who are interested in the particular details of a carotenoid or a gene, pathway or organism. To further facilitate more precise searches and comparisons, the advanced search option has been provided. The option allows the user to fetch information for two carotenoids/genes/pathways/organisms at one time.

### Tools available in the database

A tool for Structural search has been created which will be useful for the user interested in searching for carotenoids of similar or identical structure(s). We have also provided a BLAST tool for searching similar sequences in our database. Another tool for alignment and visualization has been created that will be useful for multiple sequence alignment. A self-explanatory help section was also created for providing basic information and knowhow for the database.

## Utility and discussion

### Cataloguing of information pertaining to carotenoids

**Fig. 2:**
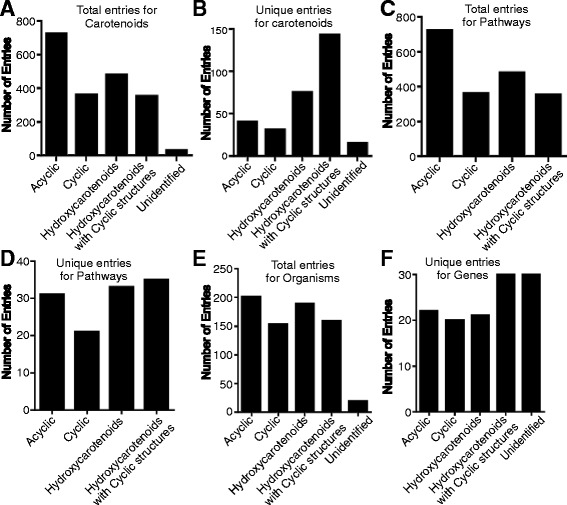
Distribution of data on the basis of (**a**) total number and (**b**) unique entries for carotenoids, total number of entries (**c**) and total entries for pathways (**d**), and distribution of carotenoids across organisms (**e**) and genes encoding for proteins involved in their synthesis (**f**). The data was divided according to the classes of carotenoids for the described aspects

The information pertaining to carotenoids, their structures, biochemical properties, genes/enzymes and the pathways involved in their biosynthesis and their distribution in prokaryotes is scattered throughout the literature and in various databases, making it difficult to study the prokaryotic carotenoids. In an effort to provide all relevant information on carotenoids and their distribution in prokaryotes, we have created this database and named it ProCarDB. For facilitating the exploration of the database, user-friendly browsing has been implemented comprehensively. The classification-based module of browsing with several useful inter-links was adopted. The browsing of ProCarDB has been implemented under the following categories (Fig. 2).

#### Carotenoid

In this category, the user can browse through different structural classes of the carotenoids along with their unique and total entries. Information on the following categories is provided: (i) Acyclic Carotenoids (with 40 unique entries and 721 total entries), (ii) Cyclic Carotenoids (with 31 unique entries and 359 total entries), (iii) Hydroxycarotenoids (with 75 unique entries and 479 total entries), (iv) Hydroxycarotenoids with cyclic structures (with 143 unique entries and 351 total entries), and (v) Unidentified carotenoids (with 15 unique entries and 29 total entries) (Fig. 2a and b). The user can obtain detailed information for each entry as a table containing information such as Molecular Weight, Molecular Formula, SMILES, IUPAC name, common name, InChI, InChIkey, chemical structures both in 2D and 3D format, UV Spectra, NMR spectra, HPLC data, etc. with their respective references.

#### Pathways

Fifty different pathways involved in the synthesis of different structural categories of carotenoids can be browsed (Fig. 2c and d). The description of each pathway in the respective organism is provided in tabulated form, wherein information pertaining to the generalized pathway, corresponding genes, and carotenoids and organisms harboring that particular pathways are provided.

#### Organism

In ProCarDB, an option for browsing organisms for different categories of carotenoids is also available. This database suggests the presence of carotenoids across 611 organisms possessing carotenoids. Carotenoids have been identified and characterized in 304 organisms. Genome analyses revealed the presence of carotenoids in another 99 organisms. Remaining, 208 organisms are either pigmented or a preliminary study has suggested the presence of carotenoids while the detailed characterization of the carotenoid is yet to be performed (Fig. 2e).

#### Taxonomy

This module is important for the taxonomist interested in analyzing the distribution of carotenoids across the prokaryotic kingdoms (Bacteria and Archaea) to the species level. This module will help them to reach a particular genera or species by following its hierarchy in the taxonomic classification. The number of carotenoids will be displayed at each taxonomic level.

#### Genes

The Genes category consists of 38 unique genes are distributed across the prokaryotes (Fig. 2f). These genes make up ~50 carotenoid biosynthesis pathways through various combinations and permutations and lead to synthesis of 304 unique carotenoids. Different genes responsible for the synthesis of different carotenoids are categorized for ease of browsing. Detailed information is also available in the form of a table containing information such as corresponding carotenoids, enzyme names, and organisms.

#### Applications of carotenoids

In this module, we have provided important notes on various applications of carotenoids. This webpage provides a link to important applications of carotenoids such as in prevention and protection from cancers, precursor of vitamin A, modulators of immune systems and as nutraceuticals. This section also describes the individual biological activity of each of the carotenoids.

### Cataloguing and extension of pathways involved in the carotenoid biosynthesis

**Fig. 3:**
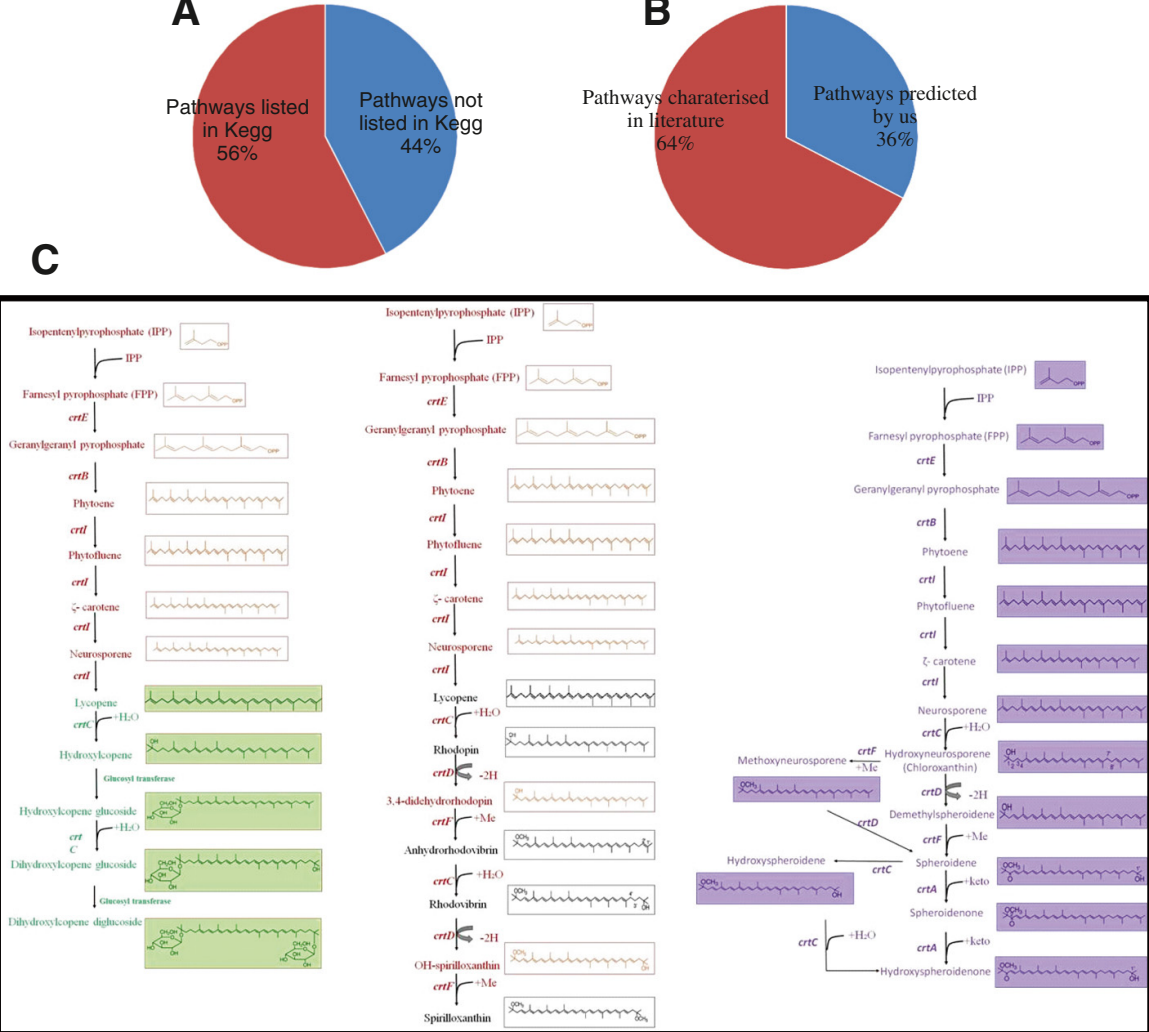
Distribution of known pathways involved in carotenoid biosynthesis. **a** A total of 50 pathways has been described in the literature. Out of these only 56 % are enlisted in the KEGG while other 44 % has not been described in the KEGG database and remains scattered in the literature. **b** We have been able to add 16 pathways based on genome mining in addition to the 33 characterized in the literature. Out of all the pathways described in this database, 64 % of pathways have been experimentally characterized while the remaining 36 % of the total enlisted pathways have been predicted based on the presence of genes in the genome, presence of carotenoids in the specific organism. **c** The color coding used in this database is as following with selected relevant examples; *Black color* represents specific steps of the pathway/specific genes described in literature but was not associated with the suggested pathway. *Green color* indicates pathway/specific steps/specific genes predicted in the literature. *Purple color* represents experimentally characterized pathway, while the *red color* represents our prediction for entire pathway/specific steps/specific genes based on genome sequence analysis/literature

Manual curation of the literature (consisting of more than 2000 published manuscripts besides books) and sequence analysis for pathways involved in the biosynthesis of carotenoids has led to the identification of 50 carotenoid biosynthesis pathways. Of these, 34 different pathways (68 % of total pathways) have been described in the literature and 16 different pathways (32 % of total pathways) were predicted by us (Fig. 3a) based on {1} the presence of carotenoid synthesis genes in the genome of an organism, and {2} carotenoids have been characterized but the pathway has not been elucidated. Basic structure of carotenoid is a long chain polyene that is synthesized using phytoene synthase that condenses C20 geranylgeranyl diphosphates. The phytoenes can afterwards undergo desaturation catalyzed by desaturases or isomerisation using isomerase or cyclization through cyclases. These derivatives could be additionally modified in numerous ways to create new carotenoids. As an illustration, Actinobacteria shows a significant diversity in carotenoid composition and utilizes several different pathways to synthesize a diversity of carotenoids. These pathways include the γ + β carotene pathway, the Isorenieratene pathway, the Canthaxanthin pathway, the Carotenal pathway, the Flavuxanthin pathway, the Sarcinaxanthin pathway, and the Chlorobactene pathway. The Zeaxanthin and Deinoxanthin pathways have been predicted for this phylum based on carotenoid genes present in the genome. Furthermore, extensive mining of literature has resulted in addition of 15 pathways to our database in addition to the 19 such pathways summarized at KEGG site. This information makes this database as the most comprehensive recourse available to the users (Fig. 3a). In this database, similar to genes, the pathways have also been color-coded for ease of understanding (Fig. 3c).

An example of extension of the pathways is the Astaxanthin pathway described in KEGG, that has been extended into a metabolic map that depicts the biosynthetic route for the formation of a number of derivatives of astaxanthin, such as 2-hydroxyastaxanthin and astaxanthin β-D glucoside [42]. Another example of an extension of the metabolic pathway is the carotenoid erythroxanthin, which is synthesized in *Brevundimonas* sp. strain SD212; its pathway has been supported experimentally [42]. However, the metabolic pathway(s) involved in the biosynthesis of erythroxanthin are not yet available in the KEGG map of carotenoid biosynthesis. Similarly, the Zeaxanthin pathway, the flavuxanthin pathway, and the bacterioruberin pathway have been studied in *Cronobacter sakazakii* [43], *Corynebacterium glutamicum* [44], and *Halobacterium salinarium* [45, 46], respectively. Although the zeaxanthin pathway is available in KEGG, there is no information for the flavuxanthin pathway or the bacterioruberin pathway. Hence, we believe that such extension of pathways involved in the carotenoid biosynthesis will be helpful to the researchers not only, to identify carotenoid intermediates but also to provide significant insights into predicting and characterizing the newer carotenoid biosynthesis pathways. These pathway-extensions make this database unique and one of the most updated information centres on the carotenoids. We have also predicted these pathways in every organism possessing carotenoids on the basis of validated or predicted pathways as published. These predictions have resulted in a tremendous increase in information and the information on pathways in each organism has been almost doubled, with approximately 33 % of pathways being predicted by us (Fig. 3b). Although these predictions need to be supported experimentally, they represent an important step forward and will tremendously help the researcher engaged in the elucidation of pathways of carotenoid synthesis.

### Identification of organisms with potential for possessing carotenoids

**Fig. 4:**
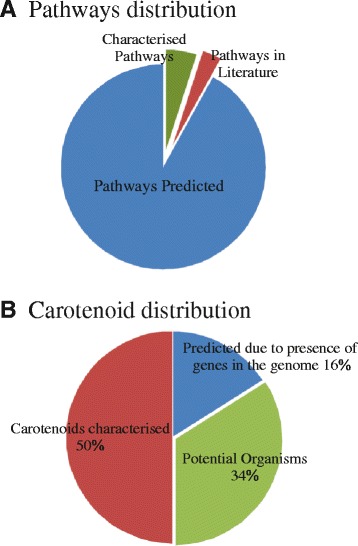
Distribution of carotenoids and their biosynthetic pathways described in this database (**a**) Distribution of carotenoid biosynthesis pathways across microorganisms based on our prediction, experimental characterization and prediction in the literature. A total of 594 organisms are characterized to have carotenoids. We have predicted pathways in 92 % of the total organism, whereas validated pathway have been illustrated in only 5 % organisms and predicted in another 3 % of total organisms. **b** Carotenoids distribution in prokaryotes. Thirty-four percent of total organisms in this database are pigmented and thus have potential for possessing carotenoids. In another 16 % of the organisms of the database, whole genome sequencing has revealed presence of genes involved in carotenoid biosynthesis, while the rest of the 50 % organisms are those in which carotenoids are characterized

A comprehensive list of prokaryotes with the potential to produce carotenoids has also been provided. Approximately 208 pigmented prokaryotes have been reported in the literature, but their carotenoids/pigments remain uncharacterized. This pool of organisms can also act as a huge potential for the research/identification of novel carotenoids. This information was further supplemented with a list of organisms that are not known to produce carotenoids, but their genome sequences have revealed the presence of genes encoding for carotenoid biosynthesis pathways. Most of them are chemotrophs and contain genes such as *crtE, crtB* and *crtI,* which together are responsible for the production of lycopene and neurosporene. Commercially, neurosporene is known for its antioxidant activity as well as protection against UV-B radiation [10], while lycopene regulates lipid metabolism and has antioxidant activity [32]. Hence, such organisms could act as a reservoir of carotenoids that have not yet been characterized. These genes could be expressed in the model organism *E. coli,* and their role in the carotenoid biosynthesis could be established. Based on the homology or experimentally identified carotenoids in the organism, we have been able to significantly extend the knowledge of carotenoid biosynthesis pathways in a large number of organisms (that constitutes 92 % of the 611 organisms that have evidence of the presence of carotenoid/s) (Fig. 4a). Whereas these pathways have been validated in only 5 % of the organisms and predicted in another 3 % of total organisms (Fig. 4a). On the other hand, presence of carotenoids has been suggested in around 450 prokaryotic organisms with no possible pathway prediction or validation. Further analysis of the distribution of carotenoids across these prokaryotes suggests that approximately 50 % of organisms possessing carotenoids have been experimentally verified, and a large number of organisms (34 %) have the potential for possessing carotenoids (A list has been mentioned in ProCarDB under the Organisms section) (Fig. 4b). Genome analysis has further suggested that another 16 % of organisms have genes required for the carotenoid biosynthesis; however, carotenoids have not been characterized from such organisms [47–49].

## Conclusions

ProCarDB is a comprehensive compilation of information pertaining to prokaryotic carotenoids. The major utility of this database is that it will help researchers’ worldwide working on carotenoids in the identification of carotenoids through its unique compilation of NMR data, UV data along with spectra, IR data, and HPLC data. This is an exclusive resource for researchers working on characterization of carotenoids. Additionally, this database assists users to find structurally similar carotenoids to their carotenoid of interest through a structure-based search tool and advance search tools. We have also provided the information about organisms and culture collections. Another important feature of this database is that it provides gene/enzyme sequences of components of pathways involved in the biosynthesis of prokaryotic carotenoids. It will be a great help for the cloning and expression of such genes in model organisms such as *E. coli*. This database is also equipped with a BLAST tool with which the users could investigate whether the genes/enzymes of interest might be involved in the biosynthesis of carotenoids. In summary, this database is a unique portal for information on carotenoids.

## Abbreviations

2D, 2 dimensional; 3D, 3 dimensional; BLAST, basic local alignment search tool; C_20_, 20 carbon unit; C_5_, five-carbon unit; CDB, conjugated double bonds; ChEBI, chemical entities of biological interest; CSS, cascading style sheets; HPLC, high-performance liquid chromatography; HTML, hypertext markup language; IJSEM, international journal of systematic and evolutionary microbiology; InChl, IUPAC international chemical identifier; IR, infrared; IUPAC, international union of pure and applied chemistry; KEGG, Kyoto encyclopedia of genes and genomes; LPSN, list of prokaryotic names with standing in nomenclature; MS, mass spectrometry; NMR, nuclear magnetic resonance; PHP, hypertext preprocessor (earlier called, personal home page); ProCarDB, prokaryotic carotenoid database; SMILES, simplified molecular-input line-entry system; UV, ultraviolet; UV-Vis, ultraviolet–visible
